# Stroke Survivors' Dependency Level and Informal Caregivers' Quality of Life in Kelantan, Malaysia: Examining the Mediating Role of Psychological Distress

**DOI:** 10.7759/cureus.64160

**Published:** 2024-07-09

**Authors:** Erwan Ershad Ahmad Khan, Wan Nor Arifin, Kamarul Imran Musa

**Affiliations:** 1 Department of Community Medicine, Universiti Sains Malaysia School of Medical Sciences, Kota Bharu, MYS; 2 Biostatistics and Research Methodology Unit, Universiti Sains Malaysia School of Medical Sciences, Kota Bharu, MYS

**Keywords:** mediation, stress, anxiety, depression, quality of life, caregiver, stroke

## Abstract

Background: Caring for stroke survivors can affect the caregiver's life, especially with the increment of patients' dependency level. This led to depression, anxiety, and stress in the caregiver, impacting their quality of life (QoL). This study aims to model relationships between caregivers' factors and stroke survivors' factors with caregivers' QoL and to estimate the mediation effects of caregivers' depression, anxiety, and stress in the relationships between stroke survivors' dependency level and caregivers' QoL.

Methods: Data from a cross-sectional study with a sample size of 250 subjects was analyzed. Linear regression was done to model the relationship between stroke survivors' factors and caregivers' factors with caregivers' QoL. Hayes's PROCESS macro model 4 for bootstrapping indirect effects was used to estimate the mediation effects of depression, anxiety, and stress in the relationship between stroke survivors' dependency level and caregivers' QoL.

Results: Stroke survivors' dependency levels that were measured by the Modified Barthel Index score (b=0.14; 95% CI: 0.07, 0.20), caregivers' depression score (b=-0.81; 95% CI: -1.44, -0.20), and caregivers' anxiety score (b=-0.73; 95% CI: -1.34, -0.12) were found to be associated with caregivers' QoL. Caregivers' depression score (effect=0.02; 95% CI for bootstrapping: 0.01, 0.04) and caregivers' anxiety score (effect=0.01; 95% CI for bootstrapping: 0.01, 0.04) were found as partial mediators in the relationship between stroke survivors' dependency level and caregivers' QoL.

Conclusion: Stroke survivors' dependency level and caregivers' depression and anxiety significantly impact caregivers' QoL, with the latter factors partially mediating this relationship. Interventions such as providing caregivers with psychological support, stress management programs, and training in caregiving skills could help mitigate these impacts and improve caregivers' QoL.

## Introduction

Informal caregivers are crucial in the post-stroke recovery process, providing practical and emotional support to stroke survivors. Often, these caregivers are family members or close friends who take on their roles out of necessity, love, or moral obligation, without formal training [1]. While the immediate physical and cognitive impacts of stroke on patients are well-studied, the effects on caregivers' QoL are just beginning to be understood [2].

Research shows a link between patients' dependency levels, measured by the Modified Barthel Index (MBI), and caregivers' QoL. As patients become more dependent on daily activities and care, the demand on caregivers increases [3]. This added burden can impact caregivers' physical, emotional, and social well-being. Therefore, healthcare providers must assess patient needs and implement supportive measures for caregivers to help maintain their QoL.

Depression, anxiety, stress, and QoL

Caring for a stroke survivor involves much more than addressing their physical needs. It includes managing medications, facilitating physical therapy, and providing continuous care [4]. This constant demand can significantly affect the caregiver's mental health, often leading to depression, anxiety, and stress. These psychological conditions can, in turn, reduce the caregiver's QoL, creating a cycle of distress and diminished well-being [5]. Understanding the mediation effect of depression and anxiety in the relationship between stroke survivors' dependency level and caregivers' QoL highlights the need for targeted interventions. Recognizing these mental health conditions as key mediators can guide the development of practical strategies such as providing psychological support, implementing stress management programs, offering training in caregiving skills, and establishing support groups. These interventions aim to reduce the psychological burden on caregivers, thereby improving their overall QoL and enhancing their ability to care for stroke survivors effectively [4,5].

Depression in caregivers often involves persistent sadness, loss of interest, and a sense of hopelessness [6]. Anxiety can manifest as constant worry and physical symptoms like increased heart rate [7]. Stress, the body's response to demands for change, can lead to feelings of being overwhelmed and burnout [8]. These psychological states not only harm the caregiver's health but also hinder their ability to provide effective care, complicating the recovery for stroke survivors [5]. Depression can diminish a caregiver's motivation and energy levels, making it difficult to maintain the consistency and patience required for effective caregiving. It can also impair cognitive functions, such as concentration and memory, which are crucial for managing the complex needs of stroke survivors. Anxiety often leads to excessive worry and fear, which can interfere with decision-making and problem-solving abilities. This heightened state of alertness can cause caregivers to become overly cautious or avoidant, potentially neglecting important caregiving tasks. Stress, characterized by a constant feeling of being overwhelmed, can lead to burnout, reducing a caregiver's ability to respond compassionately and effectively to the stroke survivor's needs. Chronic stress can also cause physical health problems, further limiting the caregiver's capacity to provide adequate care [5-8]. 

Theoretical framework

The mediation model suggests a direct positive relationship between MBI scores and the QoL experienced by caregivers. The foundation of this mediation model is anchored in Lazarus' cognitive appraisal theory of stress [9]. Adapting to this theory, the reduction of patients' dependency levels evidenced by the increment of MBI scores is expected to predict lower scores of depression, anxiety, and stress, leading to better QoL scores.

Study objectives and hypotheses

Based on this theoretical framework, the objectives of this study were to model relationships between caregivers' factors and stroke survivors' factors with caregivers' QoL and to estimate the mediation effects of caregivers' depression, anxiety, and stress in the relationships between stroke survivors' dependency level and caregivers' QoL.

Three hypotheses are proposed: In H1, caregivers' depression score negatively mediates the relationship between stroke survivors' dependency level and caregivers' QoL. In H2, caregivers' anxiety score negatively mediates the relationship between stroke survivors' dependency level and caregivers' QoL. In H3, caregivers' stress score negatively mediates the relationship between stroke survivors' dependency level and caregivers' QoL. "Negatively mediate" means that the mediator (in this case, depression, anxiety, or stress) has a negative impact on the relationship between the independent variable (stroke survivors' dependency level) and the dependent variable (caregivers' QoL). In other words, higher levels of depression, anxiety, or stress in caregivers worsen the QoL, exacerbating the negative effects of stroke survivors' dependency level on caregivers' QoL.

## Materials and methods

Reporting guideline, study design, population definitions, and study criteria

The manuscript was prepared according to AGReMA guideline [10]. This research was a cross-sectional study conducted in Kelantan, Malaysia, from January 2023 to March 2024. It focused on caregivers of stroke survivors who were receiving treatment from Neurology or Physiotherapy units at any of these four hospitals (Hospital Universiti Sains Malaysia (HUSM), Hospital Raja Perempuan Zainab II (HRPZII), Hospital Sultan Ismail Petra (HSIP), and Hospital Tanah Merah (HTM)) or through a domiciliary program by the district health offices.

The study focused on caregivers who were actively providing support to stroke survivors during the study period. The study will include caregivers who meet the following criteria: Malaysian citizenship, age 18 years or older, fluency in Bahasa Malaysia, and active involvement in the care of a stroke survivor. The stroke survivors under their care must also be 18 years or older, have a clinical diagnosis of stroke, and be receiving care from designated units or domiciliary care programs. Exclusion criteria for caregivers encompass non-residency in Malaysia, the presence of cognitive or psychiatric disorders as assessed by the research team, and households employing paid caregivers, such as nurses or domestic helpers. Caregivers with psychiatric conditions were excluded to ensure the accuracy of self-reported data regarding QoL and psychological distress, as these conditions could confound the results. Future studies might explore the impact of caregiving among this subgroup. 

Sample size estimation

Sample sizes were estimated for both objectives. For the first objective, modeling the relationship between stroke survivors' factors and caregivers' factors with caregivers' quality of life (QoL), using G*Power version 3.1.9.7 with medium effect size, f of 0.25, alpha of 0.05, power of 0.8, and the number of predictors of 19, the required sample size is 99 subjects [11]. A medium effect size was chosen as it represents a moderate relationship expected in social and behavioral sciences. An alpha level of 0.05 is a commonly accepted threshold for statistical significance, and a power of 0.8 ensures an 80% chance of detecting a true effect, balancing the risk of type I and type II errors [11]. 

For the second objective, the mediation analysis, a sample size of over 200 subjects as suggested for structural equation modeling was deemed to be adequate [12]. Hence, anticipating for 20% expected dropout rate and data entry error, a final sample size of 250 subjects was estimated.

Sampling method and subject recruitment

All eligible caregivers were recruited until the sample size of 250 was reached using convenience sampling. Caregivers were identified and recruited in two ways: when they accompanied stroke survivors to follow-up appointments at Neurology or Physiotherapy units in four hospitals (HUSM, HRPZII, HSIP, and HTM) and through domiciliary care records, which provided their contact information. The research team contacted these caregivers, and those who were interested and met the study criteria were invited to participate. To address potential sampling biases, participants were recruited from a variety of hospitals and domiciliary programs to ensure a diverse representation of caregivers. However, we acknowledge that caregivers not connected to these facilities might have different experiences. 

Research tools and data collection

Sociodemographic information for both stroke survivors and caregivers was collected using a study proforma. Caregivers' QoL was assessed with the Malay version of the Adult Carer Quality of Life Questionnaire (Malay AC-QoL), a valid and reliable tool consisting of 36 items across eight domains: support for caring, caring choice, caring stress, money matters, personal growth, sense of value, ability to care, and carer satisfaction. Items had factor loadings between 0.52 and 0.93, and composite reliability between 0.77 and 0.91. Scores range from 0 to 108, with higher scores indicating better QoL [13]. The assessment tools were translated and validated in Malay following standard procedures, including forward and backward translation, pilot testing, and psychometric evaluation, to ensure cultural relevance and reliability [13]. 

The patient's level of dependency was measured using the Malay version of the MBI, which scores from 0 (complete dependence) to 100 (full independence). This tool is reliable, with an ICC of 0.996 (95% CI: 0.995-0.997) [14]. MBI scores were obtained from caregivers through interviews.

Patient functional capabilities were assessed with the Malay version of the modified Rankin scale (mRS), which ranges from 0 (no symptoms) to 6 (death). The mRS has high reliability, with an ICC of 0.958 and a kappa statistic of 0.872 [14]. Assessments were conducted via interviews with caregivers and recorded as categorical data.

Depression, anxiety, and stress levels were evaluated using the Malay version of the DASS-21, a reliable instrument with Cronbach's alpha values of 0.84, 0.74, and 0.79, respectively [15]. Scores range from 0 to 21 for each domain, with higher scores indicating higher levels of distress. DASS-21 scores were collected from caregivers through interviews and recorded as numerical values. 

For this study, interviewers received comprehensive training on the study protocol, data collection techniques, and ethical considerations. Training sessions included mock interviews and regular supervision to ensure data consistency and reliability. 

Statistical analysis

Data was analyzed using RStudio version 2023.06.1 [16]. For the first objective, to model the relationship between stroke survivors' factors and caregivers' factors with caregivers' QoL, linear regression was conducted using the lm() function in RStudio [16]. Variables included in the multivariable linear regression model were stroke survivors' MBI score, stroke survivors' mRS score, caregivers' gender, caregivers' health problem, caregivers' depression score, caregivers' anxiety score, and caregivers' stress score, chosen for their p-values below 0.25 in initial simple linear regression analyses according to established variable selection guidelines [17,18]. A significant level was set at 0.05 as proposed by the previous guideline in determining the level of significance [19]. The two-way interaction terms between MBI score*mRS score, MBI score*caregivers' gender, and MBI score*caregivers' health problem were added to the model in order to identify modifier effects of possible moderator, to identify the conditional relationship between those variables, and to look for the need of stratified analysis as proposed by the previous guideline [18]. Next, an analysis for model fitness (R2 and F-test) was also conducted. 

**Figure 1:**
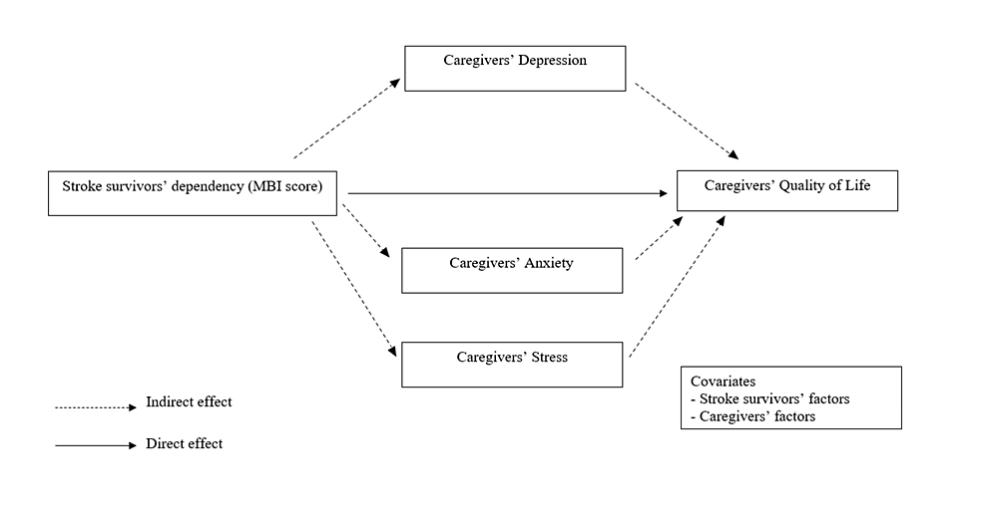
The hypothetical mediation model Image Credits: Erwan Ershad Ahmad Khan

For the second objective, the mediation analysis, the proposed model was examined in RStudio using Hayes's PROCESS macro version 4 employed for bootstrapping indirect effects [16,20]. A regression model of QoL was performed, and the stroke survivors' factors and caregivers' factors which could statistically significantly predict caregivers' QoL were selected as control variables in the mediation model (Figure 1). To test all three hypotheses, several structural equation models were employed using model 4 of PROCESS macro [20]. Hayes's PROCESS macro model 4 is ideal for mediation analysis because it simplifies the process of testing complex relationships between variables. In this study, it helps to determine whether depression, anxiety, and stress mediate the impact of caring for stroke survivors on caregivers' QoL. Model 4 is easy to implement and uses bootstrapping, which enhances the robustness and reliability of the analysis. This approach allows for the examination of whether the effects of caregiving on QoL are indirect, occurring through the mediators of depression, anxiety, and stress [20].

In the mediation models, each yielded two equations: the first examining depression, anxiety, and stress scores as the dependent variable, with stroke survivors' dependency level reflected by the MBI score as the independent variable, and the second with caregivers' QoL score as the dependent variable and all other variables (predictors and control variables) as independent. Subsequently, the direct and indirect effects of the stroke survivors' dependency level reflected by the MBI score on the QoL of caregivers were calculated. The direct effect is represented by the regression coefficient (beta) of the predictor (in this study, the MBI score) on the dependent variable, which is the caregivers' QoL. The indirect effect, meanwhile, is determined by the product of two path coefficients. "Complete mediation" occurs if the indirect effect is statistically significant while the direct effect is not. On the other hand, "partial mediation" is indicated when both the indirect and direct effects are statistically significant. If the 95% CI for the bootstrapping of the indirect effect includes zero, it signifies the absence of mediation [21]. In this study, the bootstrapping 95% CI for caregivers' stress score did include zero, while caregivers' depression score and caregivers' anxiety score did not include zero.

## Results

Table 1 presents the characteristics of stroke survivors and their caregivers within a sample size of 250. The mean age of caregivers is around 42 years, with a standard deviation (SD) of 14.59 years. The distribution by gender shows a predominance of females, who represent 163 (65.2%) of the caregivers, as opposed to males who constitute 87 (34.8%). A significant majority of caregivers are of Malay ethnicity, accounting for 235 (94%), while the remaining 15 (6%) fall into the "Others" category.

**Table 1:** Characteristics of stroke survivors and caregivers (n=250) Empty cells are intentionally left blank and are not intended to have values. SD: standard deviation; mRS: modified Rankin scale

Variables	n (%)
Caregivers' factors	
Mean age (SD)	41.96 (14.59)
Gender	
Male	87 (34.8)
Female	163 (65.2)
Race	
Malay	235 (94.0)
Others	15 (6.0)
Education level	
Secondary education	130 (52.0)
Tertiary education	120 (48.0)
Relationship with stroke survivors	
Non-spouses	167 (66.8)
Spouses	83 (33.2)
Employment status	
Employed	107 (42.8)
Unemployed	143 (57.2)
Household income	
B40	201 (80.4)
M40 and T20	49 (19.6)
Health problem	
No	181 (72.4)
Yes	69 (27.6)
Time spent on caregiving daily	
Three hours or less	61 (24.4)
More than three hours	189 (75.6)
Duration of caregiving	
Five years or less	233 (93.2)
More than five years	17 (6.8)
Caregiving training	
No	213 (85.2)
Yes	37 (14.8)
Mean depression score (SD)	3.39 (3.14)
Mean anxiety score (SD)	3.36 (3.19)
Mean stress score (SD)	3.70 (3.41)
Mean total quality of life score (SD)	85.38 (15.17)
Stroke survivors' factors	
Mean age (SD)	63.97 (10.81)
Gender	
Male	132 (52.8)
Female	118 (47.2)
Race	
Malay	235 (94.0)
Others	15 (6.0)
Mean Modified Barthel Index (SD)	55.00 (35.21)
mRS category	
1-3	118 (47.2)
4-5	132 (52.8)

The educational attainment among caregivers is balanced, with 130 (52%) having completed secondary education and 120 (48%) achieving tertiary education. Regarding their relationship with the stroke survivors, 167 (66.8%) are non-spouses, and 83 (33.2%) are spouses. The employment status reveals that 107 (42.8%) are employed, whereas 143 (57.2%) are unemployed. In terms of household income, a substantial 201 (80.4%) belong to the B40 income category, with the rest, 49 (19.6%), being part of the M40 and T20 categories. The health status of caregivers shows that a large portion, 181 (72.4%), report no health problems and 189 (75.6%) spend more than three hours daily on caregiving tasks. Most caregivers, 233 (93.2%), have been in their caregiving role for five years or less, and 213 (85.2%) have not received any form of caregiving training. Mental health assessments reveal mean depression, anxiety, and stress scores of 3.39, 3.36, and 3.70, respectively, each with an SD of around 3. The mean total QoL score for caregivers stands at 85.38, with an SD of 15.17.

For stroke survivors, the mean age is higher at approximately 64 years, with an SD of 10.81 years. Gender distribution among survivors is relatively balanced with 132 (52.8%) males and 118 (47.2%) females. The ethnicity distribution mirrors that of the caregivers, with 235 (94%) Malay and 15 (6%) in the "Others" category. The mean MBI score is 55.00, with an SD of 35.21, indicating a moderate level of dependence on caregivers for daily activities. The severity of stroke, as measured by the mRS category, is evenly split between less severe outcomes (categories 1-3), 118 (47.2%), and more severe outcomes (categories 4-5), 132 (52.8%), indicating a varied impact of stroke on survivors' functional abilities.

The linear regression analysis presented in Table 2 models the relationship between factors related to stroke survivors and caregivers and their impact on caregivers' QoL, using a sample of 250 individuals. This analysis is conducted in two stages: a crude (univariable) analysis that assesses each variable independently and an adjusted (multivariable) analysis that accounts for the simultaneous influence of multiple variables.

**Table 2:** Summary of linear regression model for caregivers' quality of life score (n=250) ^a^Simple linear regression. ^b^Multiple linear regression, F=18.90 (p<0.001), R2=0.35. No interaction was seen. Empty cells are intentionally left blank and are not intended to have values. mRS: modified Rankin scale

Variables	Caregivers' quality of life score			
	Crude b (95% CI)	P-value^a^	Adjusted b (95% CI)	P-value^b^
Caregivers' factors				
Age	-0.01 (-0.14, 0.12)	0.844		
Gender: female (vs male)	5.01 (1.08, 8.93)	0.013	3.27 (-0.06, 6.60)	0.054
Race: others (vs Malay)	-3.10 (-11.06, 4.86)	0.444		
Education level: tertiary education (vs secondary education)	-0.36 (-4.15, 3.43)	0.851		
Relationship with stroke survivors: spouses (vs non-spouses)	1.13 (-2.89, 5.14)	0.581		
Employment status: unemployed (vs employed)	-0.95 (-4.78, 2.87)	0.624		
Household income: M40 and T20 (vs B40)	-0.17 (-4.94, 4.60)	0.945		
Health problem: yes (vs no)	-4.87 (-9.06, -0.68)	0.023	-3.29 (-6.79, 0.21)	0.065
Time spent on caregiving daily: more than three hours (vs three hours or less)	0.81 (-3.60, 5.21)	0.719		
Duration of caregiving: more than five years (vs five years or less)	-2.24 (-9.75, 5.28)	0.558		
Caregiving training: yes (vs no)	-1.14 (-6.47, 4.19)	0.673		
Depression score	-2.01 (-2.56, -1.46)	<0.001	-0.81 (-1.44, -0.20)	0.010
Anxiety score	-1.83 (-2.38, -1.28)	<0.001	-0.73 (-1.34, -0.12)	0.019
Stress score	-1.41 (-1.94, -0.89)	<0.001	-0.41 (-0.97, 0.14)	0.144
Stroke survivors' factors				
Age	-0.06 (-0.23, 0.12)	0.511		
Gender: female (vs male)	1.98 (-1.81, 5.76)	0.305		
Race: others (vs Malay)	-3.10 (-11.06, 4.86)	0.444		
Modified Barthel Index	0.20 (0.16, 0.25)	<0.001	0.14 (0.07, 0.20)	<0.001
mRS category: 4-5 (vs 1-3)	-11.40 (-14.91, -7.88)	<0.001	-0.94 (-5.49, 3.61)	0.686

Significant variables as defined by a p-value of less than 0.05 were identified in the crude analysis affecting caregivers' QoL including the gender of the caregiver, with females reporting higher QoL scores compared to males; caregivers' health problems, which are associated with a decrease in QoL; depression, anxiety, and stress scores among caregivers, each negatively impacting QoL; and the MBI for stroke survivors, positively influencing caregivers' QoL. Additionally, the severity of stroke outcomes, as measured by the mRS category, is found to have a significant negative effect on caregivers' QoL.

Variables included in the multivariable analysis were caregivers' gender, health problems, and depression, anxiety, and stress scores and stroke survivors' MBI and mRS scores, selected based on p-values less than 0.25. In the multivariable analysis, the significance of caregivers' gender and health problems on QoL diminishes, indicating these factors do not have a statistically significant independent effect when other variables are considered. However, depression and anxiety scores among caregivers continue to show a significant negative association with QoL in this adjusted model. The MBI of stroke survivors remains a positive predictor of caregivers' QoL, even after adjusting for other factors. The model demonstrates a significant fit with an F-value of 18.90 and a p-value of less than 0.001 and explains 35% of the variability in caregivers' QoL scores (R-squared=0.35), suggesting that while the identified factors are important, other unmeasured variables may also influence the QoL among caregivers. No interaction between selected variables was seen.

These findings have important implications. The significant negative association of depression and anxiety with caregivers' QoL highlights the need for targeted mental health interventions to support caregivers. Programs designed to reduce depression and anxiety could potentially improve caregivers' well-being. Additionally, the positive impact of the MBI of stroke survivors on caregivers' QoL suggests that interventions aimed at improving the functional independence of stroke survivors may also benefit caregivers. Future research should explore other potential factors influencing caregivers' QoL to develop comprehensive support strategies.

Table 3 outlines the mediator effects of depression, anxiety, and stress in the dynamic between stroke survivors' dependency level, as indicated by their caregivers' QoL, with adjustments for stroke survivors' mRS scores, caregivers' gender, and caregivers' health problems. Notably, the data illustrates that higher MBI scores, signifying less dependency level of stroke survivors, positively influence caregivers' QoL through a reduction in depression and anxiety levels among caregivers. Specifically, for depression and anxiety, the analysis reports negative associations with caregivers' QoL, with significant p-values (depression: b=-0.82, p=0.010; anxiety: b=-0.73, p=0.019), meaning that increases in depression and anxiety scores are associated with declines in QoL. This highlights that as depression and anxiety levels rise among caregivers, their QoL deteriorates, underscoring the significant impact of caregivers' mental health on their well-being. Stress, as a variable, did not demonstrate a significant mediation effect within the scope of this study.

**Table 3:** Mediator effects of depression, anxiety, and stress in the relationship between stroke survivors' dependency level and caregivers' QoL, adjusted for stroke survivors' mRS score, caregivers' gender, and caregivers' health problem (n=250) Number of bootstrap samples, b = 5000. Empty cells are intentionally left blank and are not intended to have values. B: beta; CI: confidence interval; SE: standard error; t: t-test value; F: F-test value; R2: explanatory power; MBI: Modified Barthel Index; mRS: modified Rankin scale; QoL: quality of life

Variables	Outcome	b (95% CI)	SE	t	P-value
	Depression score				
MBI score		-0.02 (-0.04, -0.01)	0.01	-2.88	0.004
mRS 4-5 (vs 1-3)		0.33 (-0.78, 1.43)	0.56	0.58	0.561
Caregivers' gender: female (vs male)		-0.38 (-1.18, 0.43)	0.41	-0.92	0.356
Caregivers' health problem: yes (vs no)		0.26 (-0.58, 1.11)	0.43	0.61	0.539
F=6.83 (p<0.001), R^2^=0.10					
	Anxiety score				
MBI score		-0.02 (-0.04, -0.01)	0.01	-2.43	0.016
mRS 4-5 (vs 1-3)		0.39 (-0.74, 1.53)	0.58	0.68	0.495
Caregivers' gender: female (vs male)		0.38 (-0.44, 1.21)	0.42	0.91	0.362
Caregivers' health problem: yes (vs no)		0.47 (-0.41, 1.34)	0.44	1.05	0.293
F=5.25 (p<0.001), R^2^=0.08					
	Stress score				
MBI score		-0.01 (-0.03, 0.01)	0.01	-1.24	0.216
mRS 4-5 (vs 1-3)		0.56 (-0.67, 1.80)	0.63	0.90	0.370
Caregivers' gender: female (vs male)		0.39 (-0.51, 1.29)	0.46	0.85	0.399
Caregivers' health problem: yes (vs no)		0.28 (-0.67, 1.24)	0.48	0.59	0.557
F=2.38 (p=0.052), R^2^=0.04					
	QoL score				
MBI score		0.14 (0.07, 0.20)	0.03	4.11	<0.001
mRS 4-5 (vs 1-3)		-0.94 (-5.49, 3.61)	2.31	-0.41	0.686
Caregivers' gender: female (vs male)		3.27 (-0.06, 6.60)	1.69	1.93	0.054
Caregivers' health problem: yes (vs no)		-3.29 (-6.79, 0.21)	1.78	-1.85	0.065
Depression score		-0.82 (-1.44, -0.20)	0.31	-2.61	0.010
Anxiety score		-0.73 (-1.34, -0.12)	0.31	-2.36	0.019
Stress score		-0.41 (-0.97, 0.14)	0.28	-1.46	0.145
F=18.90 (p<0.001), R^2^=0.35					

Table 4 outlines a mediation analysis assessing the MBI's influence on QoL. The total effect of MBI on QoL is shown as 0.18, with a 95% CI between 0.11 and 0.25. The direct effect stands at 0.14 (95% CI: 0.07, 0.20), indicating the primary impact of MBI on QoL. An indirect effect of 0.04 (bootstrapped CI: 0.01, 0.07) is noted, suggesting additional mediated pathways. The analysis further explores three hypotheses concerning the mediation roles of depression, anxiety, and stress scores between MBI and QoL. It finds partial mediation through depression and anxiety scores, with indirect effects of 0.02 (bootstrapped CI: 0.01, 0.04) and 0.01 (bootstrapped CI: 0.01, 0.04), respectively. However, no mediation is observed through stress scores, with an indirect effect of 0.01 (bootstrapped CI: -0.01, 0.02). This comprehensive analysis, based on a bootstrap sample size of 5000, delineates the intricate relationships between MBI, psychological factors, and QoL.

**Table 4:** Mediation analysis summary. Number of bootstrap samples, b = 5000. Empty cells are intentionally left blank and are not intended to have values. SE: standard error; MBI: Modified Barthel Index; mRS: modified Rankin scale; QoL: quality of life

Total effect MBI->QoL (95%)	Direct effect MBI->QoL (95% CI)	Relationship	Indirect effect (bootstrapped CI)	SE	Conclusion
0.18 (0.11, 0.25)	0.14 (0.07, 0.20)	Total	0.04 (0.01, 0.07)	0.01	
		H1: MBI – depression score – QoL	0.02 (0.01, 0.04)	0.01	Partial mediation
		H2: MBI – anxiety score – QoL	0.01 (0.01, 0.04)	0.01	Partial mediation
		H3: MBI – stress score – QoL	0.01 (-0.01, 0.02)	0.01	No mediation

**Figure 2:**
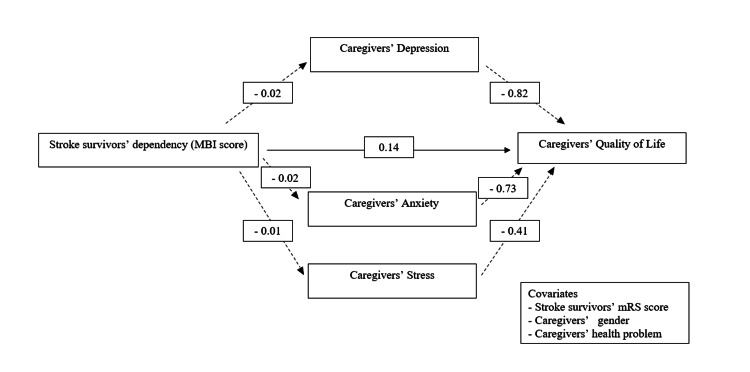
The mediation model between stroke survivors' dependency level and caregivers' quality of life Image Credits: Erwan Ershad Ahmad Khan

Based on previous results, Figure 2 illustrates the relationship between stroke survivors' dependency level, as measured by MBI score, and the QoL of their caregivers. The model visually presents these relationships with solid lines for direct effects and dashed lines for indirect effects, suggesting a comprehensive approach to understanding how stroke survivors' dependency levels can indirectly influence their caregivers' well-being through psychological stressors like depression, anxiety, and stress.

## Discussion

This study explores the relationship between the level of dependency of stroke survivors and the QoL experienced by their caregivers, using the MBI as a measure of dependency level. The MBI is a widely accepted tool that assesses the ability of an individual to perform 10 basic daily life activities, such as feeding, bathing, dressing, and mobility, assigning a score that reflects their level of independence [22]. A higher MBI score indicates a greater degree of independence. The study found a significant association between the dependency level of stroke survivors and their caregivers' QoL. Specifically, as the dependency level of the stroke survivor decreases (reflected by a higher MBI score), the QoL of the caregiver improves. This is consistent with findings from other studies that also highlight the impact of patient's dependency level towards their caregivers' QoL [23]. A lower patients' dependency level is postulated to be linked with a lesser burden for caregivers, which in turn will improve the caregivers' QoL [5]. This suggests that interventions aimed at reducing the dependency level of stroke survivors could have a positive impact on the well-being of their caregivers, highlighting the interconnectedness of patient recovery and caregiver health. At the same time, the non-significance of caregivers' gender and health problems in the adjusted analysis might indicate that their impact is mediated through other variables such as depression and anxiety. This suggests that psychological distress could be a more direct predictor of QoL than demographic factors.

In this research, the depression scores among caregivers are significantly associated with their QoL, a finding that aligns with previous research indicating a similar association [24]. Depression among caregivers often results from the chronic stress and emotional strain of caregiving responsibilities. Depression not only affects caregivers' mental health but also has tangible impacts on their physical health, social life, and financial stability, further compounding the challenges they face as found in a study elsewhere [25]. This psychological burden perhaps led to feelings of isolation, exhaustion, and helplessness, significantly diminishing a caregiver's QoL.

A higher level of anxiety among caregivers was also found to be associated with poorer QoL scores. This is consistent with findings from previous research that highlighted caregivers' anxiety as a significant predictor of their QoL [26]. Reference [26] specifically found that caregivers who reported higher anxiety levels experienced increased stress, fatigue, and emotional burden, all of which significantly lowered their overall QoL. This research underscores the importance of addressing anxiety in caregiver support programs to improve their well-being and QoL. The uncertainty and unpredictability associated with many illnesses can exacerbate these feelings, leading caregivers to feel overwhelmed and anxious about their ability to manage caregiving tasks and their own lives [27]. Possible hypotheses include that caregivers often experience anxiety due to the multifaceted challenges and responsibilities that come with caregiving. According to Denno and team in 2013, this anxiety can stem from various sources, including the emotional stress of caring for a loved one with a serious illness, financial pressures, lack of time for personal care or leisure, and concerns about the care recipient's health and future [7].

Anxiety affects caregivers' QoL in several profound ways. First, it can lead to physical health problems as chronic anxiety is associated with an increased risk of conditions like heart disease, high blood pressure, and a weakened immune system [28]. Second, it can deteriorate mental health, contributing to depression, sleep disturbances, and decreased cognitive functioning [28]. Furthermore, anxiety can hinder the caregiver's ability to provide effective care, potentially affecting the well-being and recovery of the care recipient [26]. Anxiety can also impair social relationships, as caregivers may withdraw from friends and activities they once enjoyed, leading to social isolation and deprived self-esteem [26]. This isolation can exacerbate the caregiver's anxiety, creating a cycle of stress and disconnection from essential support networks.

Strengths and limitations

This study highlights the vital link between patient autonomy and its impact on reducing caregivers' depression and anxiety, contributing insights for early intervention strategies. For example, caregivers of stroke survivors with higher MBI scores reported significantly lower levels of depression and anxiety. This finding suggests that improving patient autonomy can directly benefit caregiver mental health.

Given the aging population and the increasing need for home-based care, this research is timely in supporting caregivers, whose mental health is crucial yet often neglected. To translate these findings into practical applications, early intervention strategies should focus on enhancing patient autonomy through rehabilitation programs and assistive technologies. Additionally, implementing caregiver support groups and mental health counseling services can provide crucial emotional support and coping strategies.

Healthcare and policy changes are necessary to better support caregivers. For instance, incorporating caregiver training programs that focus on managing patient care and self-care techniques could alleviate some of the burdens. Policies that provide financial support or respite care services can also significantly reduce caregiver stress and improve their QoL. By addressing these areas, the healthcare system can become more sustainable, ultimately leading to improved outcomes for both caregivers and patients.

However, the research has limitations, such as the complex nature of measuring QoL and the challenge of generalizing findings across different caregiver groups. The study's observational design and reliance on self-reported data may also limit the clarity of the cause-and-effect relationship between patient independence and caregiver well-being. Furthermore, the complexity of caregiving dynamics and the effectiveness of proposed interventions need more exploration to ensure they are practical and beneficial across various settings. Despite these limitations, the study provides valuable insights for caregivers and healthcare providers. For caregivers, the findings highlight the importance of seeking support for mental health issues such as depression and anxiety. Healthcare providers can use this information to develop targeted interventions, such as training programs to enhance patient autonomy and support services that address caregiver mental health. To address the study's limitations and build on its findings, future research should focus on several key areas: conducting longitudinal studies to assess changes in caregiver well-being over time, including more diverse caregiver populations to enhance the generalizability of the findings, and exploring the practical effectiveness of proposed interventions in different settings. By addressing these areas, future research can provide a more comprehensive understanding of the caregiving experience and inform the development of effective support strategies for caregivers.

## Conclusions

This study demonstrates a significant link between stroke survivors' dependency level and the QoL of their caregivers, confirming the hypotheses that caregivers' depression and anxiety partially mediate this relationship. The findings reveal that as stroke survivors' dependency level decreases, caregivers experience improved QoL, primarily due to reduced levels of depression and anxiety. For example, caregivers of stroke survivors with higher MBI scores reported significantly lower levels of depression and anxiety, highlighting the positive impact of patient autonomy on caregiver mental health.

Although the study has limitations, such as challenges in generalizability, establishing causality, and reliance on self-reported data, it offers valuable insights into caregivers' needs and challenges. To better support caregivers, healthcare systems should implement regular screenings for depression and anxiety, create tailored interventions, and build supportive communities. Policymakers should use these findings to inform policies that reduce caregiver burdens.

Future research should focus on overcoming the study's limitations with longitudinal studies and a more diverse participant pool, providing a deeper understanding of caregiving's long-term effects on mental health and QoL. Additionally, exploring technological solutions could offer scalable support to caregivers, recognizing their essential role in healthcare and society. For instance, digital health tools and remote support systems could be developed to provide continuous assistance and monitoring, thus alleviating some of the burdens faced by caregivers.
